# Integrative Genomic Analyses of Patient-Matched Intracranial and Extracranial Metastases Reveal a Novel Brain-Specific Landscape of Genetic Variants in Driver Genes of Malignant Melanoma

**DOI:** 10.3390/cancers13040731

**Published:** 2021-02-10

**Authors:** Renáta Váraljai, Susanne Horn, Antje Sucker, Daniela Piercianek, Verena Schmitt, Alexander Carpinteiro, Katrin Anne Becker, Julia Reifenberger, Alexander Roesch, Jörg Felsberg, Guido Reifenberger, Ulrich Sure, Dirk Schadendorf, Iris Helfrich

**Affiliations:** 1 Skin Cancer Unit of the Dermatology Department, Medical Faculty, West German Cancer Center, University Duisburg-Essen, 45147 Essen, Germany; renata.varaljai@uk-essen.de (R.V.); Susanne.Horn@medizin.uni-leipzig.de (S.H.); antje.sucker@uk-essen.de (A.S.); alexander.roesch@uk-essen.de (A.R.); dirk.schadendorf@uk-essen.de (D.S.); 2 German Cancer Consortium (DKTK), partner site Essen/Düsseldorf, 45147 Essen, Germany; daniela.pierscianek@uk-essen.de (D.P.); guido.reifenberger@med.uni-duesseldorf.de (G.R.); ulrich.sure@uk-essen.de (U.S.); 3 Faculty Rudolf-Schönheimer-Institute for Biochemistry, University of Leipzig, 04103 Leipzig, Germany; 4 Department of Neurosurgery, Medical Faculty, West German Cancer Center, University Duisburg-Essen, 45147 Essen, Germany; 5 Institute of Anatomy, Medical Faculty, University Duisburg-Essen, 45147 Essen, Germany; verena.schmitt@uk-essen.de; 6 Department of Molecular Biology, Medical Faculty, University Duisburg-Essen, 45147 Essen, Germany; alexander.carpinteiro@uk-essen.de (A.C.); katrin.becker@uk-essen.de (K.A.B.); 7 Department of Dermatology, Medical Faculty, Heinrich Heine University, 40225 Düsseldorf, Germany; reifenbergerJ@med.uni-duesseldorf.de; 8 Institute of Neuropathology, Heinrich Heine University, 40225 Düsseldorf, Germany; joerg.felsberg@med.uni-duesseldorf.de

**Keywords:** melanoma brain metastases, matched-pair analyses, single nucleotide polymorphism, mutational load, oncogenes

## Abstract

**Simple Summary:**

Melanoma is the third most common cause of brain metastasis with a reported incidence of up to 80% leading to patients’ early mortality. Clinical activity at intracranial sites is often less and unsatisfactory when compared to extracranial metastases by using novel targeted or immune therapies. Thus, the identification of genetic alterations may provide new insights into the pathogenesis of brain metastases and this will facilitate the improvement of precision oncology. Therefore, the aim of our study was to address site-specific oncogenic alterations in intracranial metastases of 29 recurrently mutated driver genes in melanoma by next generation sequencing. In line with the branched evolution model of metastasis, we identified in our cohort of intracranial and corresponding patient-matched extracranial melanoma metastases novel genetic variants and site-specific nucleotide modifications. Therapeutic targeting of the new-identified genetic variants could help to facilitate novel, more effective therapies for prevention and/or treatment of melanoma brain metastases.

**Abstract:**

Background: Development of brain metastases in advanced melanoma patients is a frequent event that limits patients’ quality of life and survival. Despite recent insights into melanoma genetics, systematic analyses of genetic alterations in melanoma brain metastasis formation are lacking. Moreover, whether brain metastases harbor distinct genetic alterations beyond those observed at different anatomic sites of the same patient remains unknown. Experimental Design and Results: In our study, 54 intracranial and 18 corresponding extracranial melanoma metastases were analyzed for mutations using targeted next generation sequencing of 29 recurrently mutated driver genes in melanoma. In 11 of 16 paired samples, we detected nucleotide modifications in brain metastases that were absent in matched metastases at extracranial sites. Moreover, we identified novel genetic variants in *ARID1A, ARID2, SMARCA4* and *BAP1*, genes that have not been linked to brain metastases before; albeit most frequent mutations were found in *ARID1A, ARID2* and *BRAF.* Conclusion: Our data provide new insights into the genetic landscape of intracranial melanoma metastases supporting a branched evolution model of metastasis formation.

## 1. Introduction

Metastasis of malignant melanoma to the brain is a clinically challenging issue that may develop in up to 40% of patients with advanced disease [1]. The incidence of brain metastases (BM) is rising partly due to improved diagnostic techniques and advances in systemic treatment approaches directing prolonged survival of cancer patients. However, therapeutic strategies specifically targeting the metastatic cascade demonstrating survival benefit are still lacking. In consequence, metastatic spread is responsible for about 90% of cancer-related deaths across all entities [2]. Different cohort studies demonstrated cutaneous melanoma as the third most common cause of BM development [3]. BM in malignant melanoma is frequent during melanoma progression, dominating prognosis and quality of life of affected patients [4,5,6]. The incidence of BM at first presentation is about 20%, in patients suffering from advanced melanoma around 50%, and even higher as autopsy studies reported frequencies of 55 up to 80% [7,8]. Prognosis of these patients is particularly poor, resulting in median overall survival of only 17 to 22 weeks [9,10]. In consequence, the presence of BM was recently incorporated into the American Joint Committee on Cancer (AJCC) staging system as an independent prognostic factor in patients with malignant melanoma [11]. Treatment options targeting established metastases in the central nervous system (CNS) are rather limited, mainly caused by inefficient drug penetration across the blood-brain barrier (BBB). Moreover, patients with BM are commonly excluded from clinical trials, including those investigating novel targeted therapies, as the limited survival associated with BM prevents reaching study endpoints.

Large-scale sequencing studies have revealed the widespread genetic diversity of melanoma, directing therapy efficacy and patient’s survival [12,13,14]. Thus, in the current era of molecularly targeted therapies and personalized medicine, therapeutic decisions are increasingly being tailored according to the individual genetic profile of a tumor. Primary melanomas and extracranial metastases have been extensively studied, but largely due to lack of available tissue, the biology of intracranial melanoma metastases remains poorly understood. However, genomic sequencing of primary tumors and intracranial metastases from different types of solid cancers revealed remarkable genetic heterogeneity but also further genetic modifications from primary tumors to metastases at distant sites, or BM specific mutations, implicating that intracerebral metastases develop from “branched genetic evolution” [15,16,17,18].

Several large-scale sequencing studies in melanoma identified significantly mutated melanoma genes, such as *NF*), *ARID2*, *TERT* or *RAC* beside the well-established oncogenes including *NRAS*, *BRAF* or *KIT* [13,19,20,21]. About half of the melanoma patients harbor activating mutation in oncogenes involved in the mitogen-activated protein kinase (MAPK) pathway, e.g., *BRAF* or *NRAS* and/or constitutive activation of the cell cycle modulating phosphoinositide 3-kinase (PI3K) signaling pathway. Combining BRAF with MEK inhibitors shows meaningful clinical efficacy in terms of objective response rates in extracranial melanoma [22] and quite recently, also in intracranial activity with response rates of 44 to 59% in patients with melanoma BM. The median intracranial duration of response was 6.5 month in a prospective study in melanoma patients [23]. However, integrative genetic analyses focusing on oncogenic alterations of the “hotspot” cancer genes of anatomically and temporally distinct melanoma metastases from the same patient are still limited. These analyses are of pivotal clinical importance for the understanding of the metastatic seeding of cancer cells to the brain, but also to guide the development of next generation targeted therapies.

The aim of this study was to identify BM-associated driver mutations in melanoma with potential for translation into future therapeutic strategies. Therefore, we performed targeted next-generation sequencing of 29 melanoma-associated genes in intracerebral melanoma metastases and “matched” melanoma metastases from extracranial sites.

## 2. Results

### 2.1. Patient Characteristics

We included 47 patients, 20 females and 27 males, diagnosed with malignant cutaneous melanoma. The mean age at the time of first diagnosis of malignant melanoma was 60 ± 14 and 55 ± 14 (mean ± SD), respectively (incomplete clinical data from 14 patients). The time of first intracranial tumor presentation occurred at 56 ± 14 (mean ± SD) years in the whole cohort. From 16 of the 47 patients matched biopsies were available from intracerebral and extracranial metastases allowing for comparative genetic analyses. In total, we included 54 melanoma metastases with cerebellar (5 cases), cerebral (48 cases) or spinal (1 case) localization. The set of 18 extracranial metastases included 16 cutaneous, 1 lymph node and 1 adrenal gland melanoma metastases. Relevant clinical data of these patients are listed in Table 1.

### 2.2. Comparison of Brain Metastases and Metastases of Other Sites

**Figure 1:**
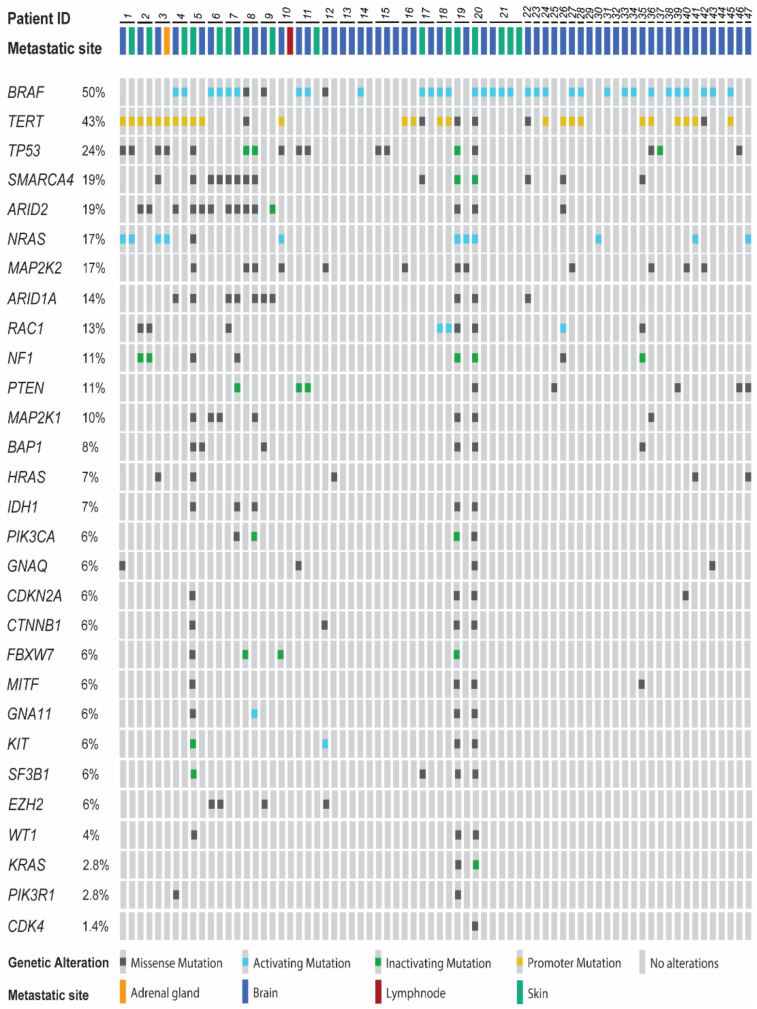
OncoPrint chart displaying the type of detected genomic alterations in the 29 gene-panel across 72 sequenced melanoma metastases. Patient identification (ID) numbers are given. Each column represents a sample, sample type is indicated at the top in a color-coded manner. The percentage of samples with detectable alteration is listed on the left for each gene.

We called single nucleotide variants including known mutations and rare single-nucleotide-polymorphisms (SNPs) in 47 of 54 intracerebral and in 15 of 18 extracranial metastases (Figure 1). To evaluate co-occurrence and mutual exclusivity of the detected variants, we tested sample pairs between the 29 genes as shown on the OncoPrint. We evaluated genes that were altered in at least 10% of the samples (*n* ≥ 7). SMARCA4 mutations significantly co-occurred with ARID2 (*p* = 0.001, log_2_ odds ratio [OR] >3, *n* = 9) and ARID1A (*p* < 0.0001, log_2_ OR > 3, *n* = 7) mutations. Similarly, TERT mutations significantly co-occurred with NRAS and MAP2K2 (both: *p* = 0.046, log_2_ OR = 2.373, *n* = 9), RAC1 (*p* = 0.004, log_2_ OR > 3, *n* = 8), and NF1 (*p* = 0.035, log_2_ OR > 3, *n* = 7) mutations. ARID2 was mutated in samples that also carried ARID1A (*p* < 0.0001, log_2_ OR > 3, *n* = 8) or NF1 mutations (*p* < 0.0001, log_2_ OR > 3, *n* = 8). Finally, TP53 mutations were highly likely present in NRAS mutated samples (*p* = 0.005, log_2_ OR > 3, *n* = 8).

**Figure 2:**
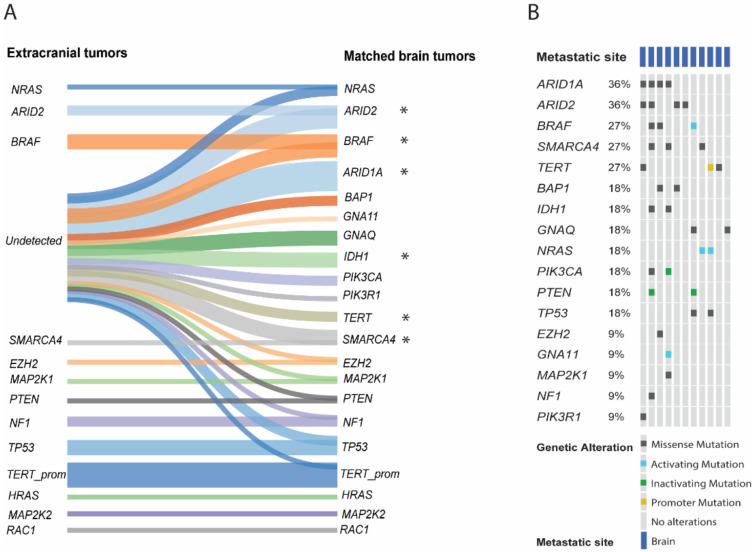
(**A**) Sankey diagram illustrating the shared and unique variants in 20 genes across 11 matched samples (left: extracranial, right: brain tumor tissues). Variants that were detected in extracranial, but not in brain metastases are not shown. Asterisk indicate *p* ≤ 0.05 significance from Chi-square test, where the proportion of samples with shared and unique variants were tested (extracranial vs. BM vs. both). (**B**) OncoPrint chart displaying the frequency of detectable alterations in 17 genes that were specific to brain tissues.

The 16 matched biopsies allowed for the direct comparison of anatomically and temporally distinct melanoma metastases. In 11 of the 16 pairs, we detected genetic variants in BM that were absent in the corresponding metastases obtained from other sites of the body. These included mutations in 12 well-known cancer genes associated with cutaneous and/or uveal melanomas: BRAF, NRAS, PIK3CA, TERT, IDH1, EZH2, NF1, PTEN, TP53, MAP2K1, GNAQ and GNA11. Interestingly, we identified variants in five new candidate genes, i.e., ARID1A, ARID2, SMARCA4, PIK3R1 and BAP1, which neither have been previously identified in melanoma BM nor reported in CNS metastases from other cancers (Figure 2A). We assessed the progression patterns of matched extracranial and BM samples to determine which gene variants are significantly associated with BM within our matched sample dataset. Variants in 6 genes showed a statistically significant occurrence specific to BM. Mutations in ARID1A (*p* = 0.0205), ARID2 (*p* = 0.0207), BRAF (*p* = 0.023) SMARCA4 (*p* = 0.015), TERT (*p* = 0.0041), and IDH1 (*p* = 0.0041) were significantly exclusive to BM while the same variants remained undetected in the corresponding extracranial tumor tissues.

Next, we analyzed the mutation frequency in the mutational driver genes. Overall, the most frequently mutated genes in BM included ARID1A, ARID2, BRAF, and SMARCA4 (Figure 2B, Table 2). ARID1A variants (P650S, P1568S, L2119S, P1562L, G831A, G423E4) and ARID2 variants (D239N, S1489L, P1022S, S297F) were detected in 4 intracerebral melanoma tissues. BRAF variants (P336L, G73E and V207E) and SMARCA4 variants (P262L, P919S, E1113K, H884Y) were detected in 3 melanoma BM. We also detected nucleotide transitions resulting in gene activation in NRAS (Q61K, Q61R), in the promoter region of TERT (chr5:1295250 G > A), and in GNA11 (R183C), the latter being frequent in uveal melanoma [24] and primary meningeal melanocytic tumors [25]. Moreover, recurrence was seen for three brain-specific variants, in BRAF (V207E), and TP53 (E171G) in 2 of 16 tested pairs. These genetic variants include a typical cancer driver mutations in BRAF. Brain-specific loss-of-function mutations were detected in PIK3CA (Y1157*) and in PTEN (Q219*, A328fs). Previously not described variants in PIK3R1 (S43R, F41C) were detected private to BM. The detailed description of these variants are reported in Table 2. Furthermore, we evaluated whether some mutations tend to co-occurred in BM. SMARCA4 significantly co-occurred with IDH1 and PIK3CA mutations (both: *p* = 0.042, log_2_ OR > 3, *n* = 2). As seen before, SMARCA4 tended to co-occurred with ARID1A and ARID2 mutations, however, here significance was not reached (*p* = 0.083 and *p* = 0.583, respectively). IDH1 mutations were highly likely present in samples that also carried PIK3CA mutations (*p* = 0.042, log_2_ OR > 3, *n* = 2). We also searched in 180 different cancer sequencing studies (including over 47000 samples using the cBioPortal site) for evidence of BM associated variants, and while all of the detected variants in the 17 genes were found in different cancer types, only 4 variants (NRAS Q61, TP53 E171G, PTEN A328*, ARID2 S297F) were detected corresponding to CNS cancers (*n* = 10 tumor tissues). The presence of these mutations in primary CNS tumors further support their significance in brain metastases.

### 2.3. UV Mutation Signature

**Figure 3:**
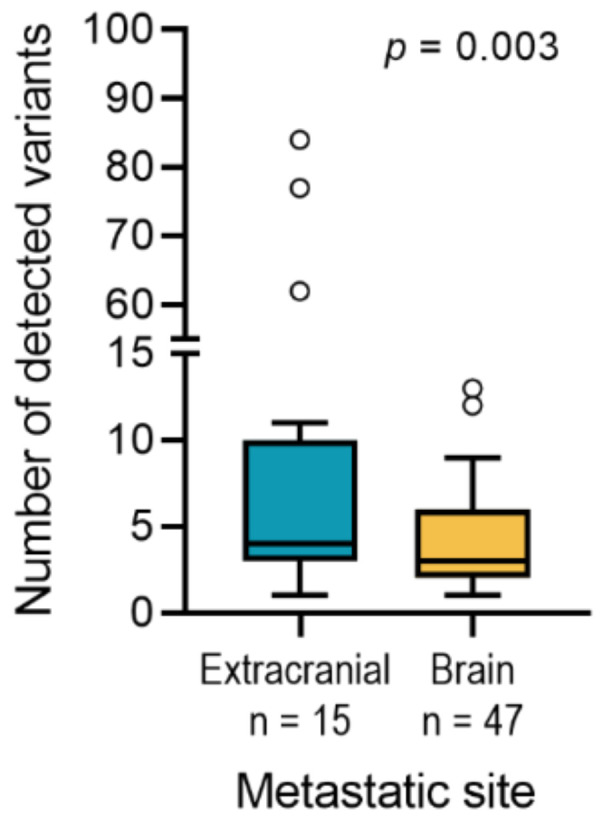
Box and whiskers plot showing the sum of called variants in extracranial and brain meta-static tissues including unpaired samples. The number of detected variants were summed and plotted in extracranial metastases (mean: 17, median: 4, range: 1–83) and in brain metastases (mean: 4, median: 2, range: 1–12). *p*-value indicates two-sided unpaired *t*-test.

Metastases located in sun-exposed areas of the skin might accumulate more mutations compared to metastases located of sun-protected areas of the body, such as metastases of the CNS. Indeed, using our multi-gene amplicon panel the number of called SNVs in BM was significantly lower compared to metastases at other sites (*p* = 0.003, Figure 3).

**Figure 4:**
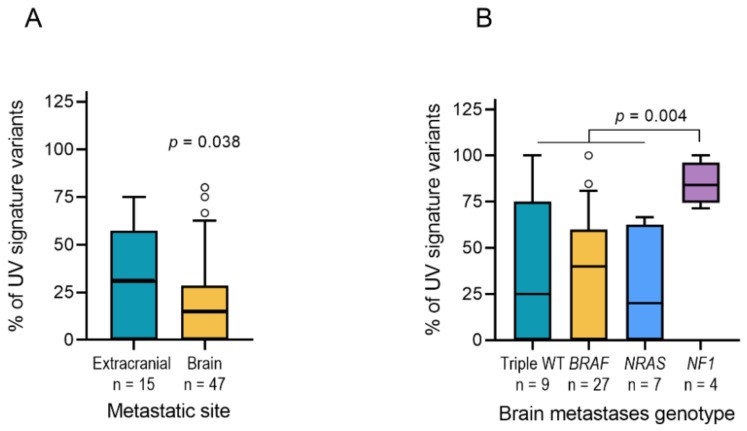
(**A**) Box and whiskers plot showing the frequency of C > T/G > A UV mutation signature in extracranial and brain metastatic tissues including unpaired samples. *p*-value indicates two-sided unpaired *t*-test. (**B**) Box and whiskers plot showing the frequency of C > T/G > A UV mutation signature across mutational subtypes of brain metastases. *p*-value from two-sided unpaired *t*-test.

In addition, large-scale genome sequencing indicated that considerable elevated baseline mutation rates in cutaneous melanoma are primarily represented by increased abundance of cytidine to thymidine (C > T or CC > TT) transitions that are characteristic for an UV-light-induced mutational signature [13,26,27]. As most of the metastases at extracranial sites were indeed cutaneous metastases (16/18), we analyzed the frequency of C > T transitions to address confounding impact of high mutational load due to UV mutagenesis. As expected, BM showed less frequent (C > T) variants than extracranial metastases (*p* = 0.038, Figure 4A). Next, we analyzed C > T transitions in four mutational subtypes of melanoma, BRAF-, NRAS-, NF1-mutant samples and triple-wildtype samples [28]. As reported, NF1-mutant samples showed a significantly higher abundance of C > T transitions when compared to samples from the other three subgroups (*p* = 0.004, Figure 4B).

## 3. Discussion

Melanoma is the third common cause of intracerebral metastases after lung and breast carcinoma [29]. In recent years, treatment of melanoma has made remarkable progress in the era of precision medicine and targeted oncology. However, treatment of BM in melanoma patients remains one of the major clinical challenges. Although recent data show therapeutic activity against melanoma BM by targeting the *BRAF* pathway or by immune checkpoint inhibition using inhibitors against the cytotoxic T-lymphocyte-associated Protein 4 (CTLA-4) or the Programmed cell death protein 1 (PD-1), response rates are rather limited and, overall, patients still have a poor prognosis [30,31]. Intracranial response rates for *BRAF* inhibitors in patients harboring mutant *BRAF* (V600E) have been reported in the range of 30–50%, with an increased response in asymptomatic patients and patients without previous local BM therapy [10,23,32]. However, while stable disease was achieved in the majority of patients with partial or complete response, CNS progression has been reported in almost 75% of patients undergoing BRAF-targeted therapy [33,34].

Furthermore, recent data indicate activity of anti-CTLA-4 [35] and anti-PD-1 monotherapy [36,37], or a combination of both, resulting in intracranial response rates of up to 47 %; however, the level of intracranial response was not translated into improved overall survival [38]. Thus, there is a time-critical clinical need to understand the biology of BM and their molecular signatures to guide the development of novel therapeutic approaches against metastases of the CNS.

The divergence of genetic alterations detected in multiple clinical sequencing projects including primary tumors and “patient-matched” BM raised the notion, that each distinct lesion might harbor a unique set of oncogenic alterations. Collecting patient-matched tissue sets is a major challenge mainly due to the fact that resection of BM is performed by neurosurgeons while primary melanomas and cutaneous melanoma metastases are resected by dermatologists, often in private practice. Nevertheless, in this study we were able to screen 54 melanoma brain metastases of 47 patients and 16 matched extracranial melanoma metastases collected from 16 corresponding patients to focus on genetic variants in anatomically and temporally distinct metastases. We focused on 29 genes, identified for their cancer driving capability in cutaneous or uveal melanoma. In line with the literature, we found a reduced mutation load in tumor samples from intracranial localization, including less frequent UV-light-induced mutational signatures when compared to extracranial, mostly cutaneous metastases. In a recently published study by Fischer et al., [7] the relevance of mutations were also evaluated in patient matched extracranial and BM samples from 17 patients across 74 genes. While only 16 genes overlapped between our gene panel and the gene panel by Fischer et al., in 5 of those genes (*BRAF*, *GNAQ*, *NF1*, *BAP1*, *PIK3CA*, *PIK3R1*) mutations private to BM were confirmed. Although that the exact nucleotide transitions within those genes could not be directly compared to the variants disclosed in our study, the clinical significance of distinct genetic BM alterations is further supported. Notably, 56% of our “matched-pair” metastases presented genetic variants private to BM. Moreover, we detected single nucleotide transitions in *ARID1A*, *ARID2*, *SMARCA4* and *BAP1*, all genes which have not been associated before with CNS metastases of melanomas. However, beside modifications in *ARID1A*, genetic variants in *BRAF* were detected most frequently compared to alterations in other genes. Point mutations in *BRAF*, mostly resulting by the substitution of valine for glutamin acid at codon 600 (VAL600 Glu or V600E) but also NRAS are the most frequent activating somatic events in melanoma and have been extensively studied in large cohorts of clinical trials for their impact on disease progression and therapeutic intervention [13,23,38,39,40,41]. In the metastatic situation, mutations in both genes were implicated in worse survival outcomes and increased risk for development of BM [9;10]. In line with previous studies of extracranial melanomas, none of our 16 sample pairs presented coexistence of *BRAF* and *NRAS* mutations. However, the clinical correlates of *BRAF* and *NRAS* mutations in melanoma BM are limited. Fang and colleagues recently described the relationship between tumor *BRAF*/*NRAS* mutation status, clinical characteristics and response to conventional therapy in patients with melanoma BM [42]. The authors resumed that either an activating *BRAF* mutation or an *NRAS* mutation significantly increases the local failure rate compared to patients who carried tumors that were wildtype for both genes.

*ARID1A* encodes the ATPase subunit interacting component BAF250, which, together with a set of core subunits, represents the switch/sucrose non-fermentable (SWI/SNF) chromatin remodeling machinery, providing access of proteins to DNA [43]. *ARID1A* mutations were first reported in ovarian clear cell carcinoma and subsequently described in endometrium-derived carcinomas [44,45]. However, growing evidence indicated that *ARID1A* may have a widespread suppressive role in various cancer entities [46,47]. In line with this finding, it has been reported that *ARID1A* negatively regulates *TERT* transcriptional regulation and activity via binding to *TERT* regulatory elements. In consequence, cells showed a repressive histone mode and a survival advantage via telomere maintenance [48]. Moreover, recent data reported by Shen and colleagues correlate loss-of-function mutations in *ARID1A* with the efficacy of anti-PD1 therapy, as a result of enhanced intratumoral lymphocyte recruitment and up-regulation of PD-L1 in preclinical models of ovarian cancer [49]. However, the clinical significance of such differential expressions and the function of the *ARID1A* protein remain undefined due to the lack of studies using fresh human tumor samples. In addition to *ARID1A* mutations, our data also indicated variants of unknown significance in other components of the SWI/SNF complex, such as *ARID2* and *SMARCA4*. Single nucleotide modifications in *ARID2* and *SMARCA4*, all of unknown function but together with mutated splice site and frameshifts events often summarized and reviewed as “loss-of-function mutations”, have already been reported in extracranial melanomas [13,50,51,52]. In summary, 11/54 (20%, Figure 2A) and 6 of the 11 patient-matched brain pairs (55%, Figure 2B) of our discovery samples harbored genetic modifications in a component of the SWI/SNF machinery, implicating a potential role for dysregulation of chromatin remodeling in promoting the formation of melanoma CNS metastases. Interestingly, *SMARCA4*, first identified to be highly mutated in NSCLC patients [53], can act as a corepressor of *ZEB1*, promoting epithelial-to-mesenchymal transition (EMT) [54]. EMT is a major signature of highly tumorigenic cancer cells, inducing E-cadherin mediated disruption of cell-cell contacts and, consequently, metastatic dissemination of cancer cells to distant organs [55]. In line with this finding, RNASeq data of spontaneously growing brain metastases of our novel preclinical melanoma models but also human BM biopsies clearly indicate strong EMT signatures when compared to RNASeq profiles of extracerebral melanoma metastases).

In 2 of 16 paired patient samples, we found mutations of unknown relevance in *BAP1* which were absent in the corresponding extracranial metastases. In both cases, we detected single nucleotide changes A > T or C > T in position 52436829 (protein change V295M and L650H). Germline pathogenic variants and somatic mutations in the *BAP1* gene have been described in ocular and cutaneous melanoma and paralleled with a highly aggressive ocular melanoma phenotype [56,57] but also detected in several other cancer subtypes [58].

In summary, our data support the concept that melanoma metastasis formation is frequently driven by branched-evolution with unique gene modifications restricted to intracranial metastases and absent in corresponding metastases of distant sites. However, larger cohorts of matched primary and metastatic tumors at different sites as well as large-scale genomic sequencing are needed to comprehensively elucidate the landscape of genetic alterations in melanoma brain metastases and uncover their clinical impact.

## 4. Materials and Methods

### 4.1. Patient Material

Patients suffering from advanced skin melanoma were included in this study. Samples of intracranial melanoma metastases were provided by the tissue banks at the Department of Neurosurgery, University Hospital Essen, as well as the Institute of Neuropathology, Heinrich Heine University Düsseldorf, Germany. Extracranial metastases of corresponding patients (“matched-pair” samples), including cutaneous, lymph node and adrenal gland melanoma metastases, were retrieved from the Skin Cancer Biobank (SCABIO) of the Department of Dermatology, University Hospital Essen, or the Department of Dermatology, Heinrich Heine University Düsseldorf, Germany. Intracranial and extracranial melanoma metastases were histopathologically diagnosed by our in-house neuropathologists (for intracranial metastases) and dermatopathologists (for extracranial metastases). Informed patient consent and the appropriate IRB approval was obtained for all patients. The study was performed with approval by the ethics committee of the Medical Faculty, University Duisburg-Essen (ethics approvals no. 11-4715 and no. 15-6723-BO), and the ethics committee of the Medical Faculty, Heinrich Heine University Düsseldorf (ethics approval no. 5246).

### 4.2. DNA Isolation

Sections of 10 µm thickness (3–4 sections per sample) were cut from fresh frozen samples of CNS metastases or cutaneous, lymph node and adrenal gland metastases of patients diagnosed with malignant melanoma. Prior DNA extraction standard hematoxylin and eosin (H&E) staining was performed for visualization of the tissue morphology. After histopathological confirmation, the tumor area was marked as “Region of Interest (ROI)” by our inhouse pathologists and manually dissected from the slide. Samples from intracranial and extracranial origin presenting a tumor content of >80% in the ROI have been included in the analysis. Genomic DNA was isolated by using the QIAmp DNA Mini Kit (Qiagen, Hilden, Germany), including RNase A treatment, according to the manufacturer’s instructions.

### 4.3. Targeted Sequencing

A custom designed amplicon-based sequencing panel covering 29 genes known as being recurrently mutated in cutaneous and uveal melanomas was applied [59]. Sequencing libraries were prepared applying the GeneRead Library Prep Kit from Qiagen^®^ according to the manufacturer’s instructions. For adapter ligation and barcoding samples, we applied the NEBNext Ultra DNA Library Prep Mastermix Set and NEBNext Multiplex Oligos for Illumina from New England Biolabs. Twelve to 24 samples were sequenced in parallel on an Illumina MiSeq next generation sequencer.

Sequence analysis was performed with CLC Cancer Research Workbench (Qiagen^®^). In brief, the following steps were applied. The CLC workflow included adapter trimming and merging paired reads before mapping to the human reference genome (hg19). Insertions and deletions as well as single nucleotide variants were detected, local realignment and primer trimming followed. Additional information was then obtained regarding potential mutation type, single nucleotide polymorphisms with a minor allele frequency of 1 percent and higher, and conservation scores by cross-referencing databases (COSMIC, ClinVar, dbSNP, 1000 Genomes Project, HAPMAP and PhastCons-Conservation_scores_hg19). After CLC processing, csv files were analyzed manually. Mutations affecting protein coding regions of genes were regarded if predicted to result in non-synonymous amino acid changes. Questionable low frequency background mutations calls, not uncommon in FFPE amplicon sequencing approaches were excluded [60] by applying the following thresholds: an overall coverage of the mutation site ≥ 30 reads, ≥15 reads reporting the mutated variant and ≥5% frequency of mutated reads. Genetic variants private to BM were reported only when the target region was covered sufficiently as described above in the corresponding metastasis of extracranial origin.

### 4.4. Data Analysis

To confirm validity of the analyzed genomic variants, we checked for pathogenic score in ClinVar [61] and in COSMIC databases [62]. Genomic variants were furthermore searched in 180 different cancer sequencing studies including over 47000 samples using the cBioPortal website [63]. OncoPrint maps were generated using the OncoPrinter tool [64,65]. Sankey diagram was created using the SankeyMATIC tool. Two-sided unpaired *t*-test was used for the determination of statistical significance between experimental groups. Mutational co-occurrence or mutual exclusivity was tested by two-sided Fisher’s exact test with multiple test correction. Mutational association with organ site (extracranial vs. BM vs. both) was analyzed by two-sided Chi-square test. Chi-square *p* value was reported only for those genes where a valid comparison could be made i.e., all groups (extracranial vs. BM vs. both) contained values > 0. *p* values ≤ 0.05 were considered as statistically significant.

## 5. Conclusions

Melanoma brain metastases demonstrate site-specific genetic variants (62%). Mutations in ARID1A, ARID2, BRAF, SMARCA4, TERT and IDH1 were significantly exclusive to intracranial metastases while the same variants remained undetected in the corresponding extracranial tumor tissues. We also detected previously not described genetic variants in the “hot spot” oncogenic driver genes private to melanoma brain metastases. Analyses of large cancer sequencing data sets of primary CNS tumors point the significance of our novel variants for tumor establishment in the brain.

## Figures and Tables

**Table 1:** Patient characteristics.

Characteristics	Number of Cases	Age at Diagnosis [Years ± Range]	Age at Surgery [Years ± Range]
Total patients	47		
Sex			
Male	27	55 ± 14	59 ± 14
Female	20	60 ± 14	60 ± 11
Metastases	72		
Brain	54	56 ± 14	57 ± 14
Skin	16	53 ± 20	55 ± 1
Lymph node	1	44	45
Adrenal gland	1	47	47
Localization of BM			
Cerebellar	5	-	-
Cerebral	48	-	-
Spinal	1	-	-
Therapy			
Mono-CT	2		
Mono-RT	4		
Mono-IMT	2		
CT + RT	1		
CT + IMT	3		
RT + IMT	5		
Unknown	30		
Status		-	-
Alive	0	-	-
Dead	45	-	-
Lost to follow up	2	-	-

BM: brain metastasis, CT: chemotherapy, RT: radiotherapy, IMT: immunotherapy.

**Table 2:** Variants specific to brain-metastases of melanoma.

Pair	Gene	Chr	Region	Cov	Freq (%)	Subs	Protein Change	Relevance
9	EZH2	7	148523587	82	14.6	C > T	C289Y	Missense-US
9	ARID1A	1	27087374	813	35.6	C > T	P650S	Missense-US
9	ARID1A	1	27101420	266	5.6	C > T	P1568S	Missense-US
9	BAP1	3	52439829	90	32.2	C > T	V295M	Missense-US
9	BRAF	7	140494241	2446	5.2	G > A	P336L	Missense-US
8	SMARCA4	19	11097605	378	39.2	C > T	P262L	Missense-US
8	MAP2K1	15	66727441	1118	48.3	T > A	F53I	Missense-US
8	ARID1A	1	27106745	10123	57.1	T > C	L2119S	Missense-US
8	IDH1	2	209106800..209106801	223	41.3	GG > AA	A212V	Missense-US
8	PIK3CA	3	178951900	330	43.3	C > A	Y1157*	inactivating
8	GNA11	19	3115011..3115012	130	40.8	CC > TT	R183C	activating
2	TERT	5	1255474	148	5.4	G > A	P1029S	Missense-US
7	IDH1	2	209108164	229	5.2	C > T	E229K	Missense-US
7	BRAF	7	140481411	796	12.8	C > T	G73E	Missense-US
7	ARID1A	1	27101403	513	6.0	C > T	P1562L	Missense-US
7	ARID1A	1	27089536	686	6.1	G > A	G831A	Missense-US
7	SMARCA4	19	11132539	363	10.2	C > T	P919S	Missense-US
7	PIK3CA	3	178927423	111	6.3	C > T	L396F	Missense-US
7	NF1	17	29560040	485	6.4	C > T	L1173F	Missense-US
7	NF1	17	29663403	412	5.3	C > T	T1999I	Missense-US
7	SMARCA4	19	11138581	95	12.6	G > A	E1113K	Missense-US
7	ARID2	12	46230381	91	8.8	G > A	D239N	Missense-US
7	IDH1	2	209113246	216	5.1	C > G	K87N	Missense-US
7	PTEN	10	89717630	115	6.1	C > T	Q219*	inactivating
5	BAP1	3	52436829	139	5.8	A > T	L650H	Missense-US
5	ARID2	12	46246372	377	39.0	C > T	S1489L	Missense-US
6	ARID2	12	46244970	1795	25.5	C > T	P1022S	Missense-US
10	NRAS	1	115256529	378	43.4	T > C	Q61R	activating
10	TP53	17	7578389	440	38.2	G > A	R142C	Missense-US
10	TERT promoter	5	1295250	324	33.0	G > A	C250T	activating
11	TP53	17	7578418	444	93.5	T > C	E171G	Missense-US
11	BRAF	7	140453136	124	54.0	A > T	V207E	activating
11	TP53	17	7578418	2332	28.1	T > C	E171G	Missense-US
11	GNAQ	9	80537098..80537099	171	6.4	TG > GA	P100L	Missense-US
11	PTEN	10	89720833	224	29.0	A > -	A328fs	inactivating
11	BRAF	7	140453136	906	23.2	A > T	V207E	activating
1	GNAQ	9	80537113	420	9.3	G > C	D95E	Missense-US
1	GNAQ	9	80537098..80537099	218	17.4	TG > GA	P100L	Missense-US
3	NRAS	1	115256530	370	41.9	G > T	Q61K	activating
3	SMARCA4	19	11132434	1563	28.9	C > T	H884Y	Missense-US
4	TERT	5	1254594	294	34.4	C > T	A1062T	Missense-US
4	ARID1A	1	27056272	758	35.0	G > A	G423E	Missense-US
4	PIK3R1	5	67522632	181	7.2	T > G	S43R	Missense-US
4	PIK3R1	5	67522628	182	7.7	T > G	F41C	Missense-US
4	ARID2	12	46230641	749	24.0	C > T	S297F	Missense-US

Brain specific variants discovered in 11 matched sample pairs. Chr: Chromosome, Cov: Coverage; Freq: Frequency of variant in sequencing reads, Subs: Substitution; AA: Amino acid, NA: Not available; US: unknown significance.

## Data Availability

Data will be shared upon request.

## Abbreviations

AJCC: American Joint Committee on Cancer; ARID1: AT-rich interactive domain-containing protein 1; ARID2: AT-rich interactive domain-containing protein 2; BBB: blood-brain barrier; BM: brain metastases; BRAF: v-Raf murine sarcoma viral oncogene homolog B; CNS: Central Nervous System; CTLA-4: cytotoxic T-lymphocyte-associated Protein 4; EMT: epithelial-to-mesencymal transition; EZH2: Enhancer of zeste homolog 2; GNA11: G Protein Subunit Alpha 11; GNAQ: Guanine nucleotide-binding protein G(q) subunit alpha; IDH1: Isocitrate dehydrogenase 1; KIT: cluster of differentiation 117; MAPK: mitogen-activated protein kinase; MEK: Mitogen-activated protein kinase kinase; NF1: Neurofibromin 1; NRAS: Neuroblastoma RAS viral oncogene homolog; NSCLC: Non-small cell lung cancer; PI3K: phosphoinisitide 3-kinase; PD-1: Programmed cell death protein 1; PIK3CA: Phosphatidylinositol-4,5-Bisphosphate 3-Kinase Catalytic Subunit Alpha; PTEN: Phosphatase and tensin homolog deleted in from chromosome ten; RAC: Ras-related C3 botulinum toxin substrate; SD: Standard deviation; SWI/SNF: switch/sucrose non-fermentable; TERT: Telomerase reverse transcriptase, ZEB1: Zinc Finger E-Box Binding Homeobox 1.
